# Structure-function coupling as a correlate and potential biomarker of cognitive impairment in multiple sclerosis

**DOI:** 10.1162/netn_a_00226

**Published:** 2022-06-01

**Authors:** Shanna D. Kulik, Ilse M. Nauta, Prejaas Tewarie, Ismail Koubiyr, Edwin van Dellen, Aurelie Ruet, Kim A. Meijer, Brigit A. de Jong, Cornelis J. Stam, Arjan Hillebrand, Jeroen J. G. Geurts, Linda Douw, Menno M. Schoonheim

**Affiliations:** Department of Anatomy and Neurosciences, Amsterdam UMC, Vrije Universiteit Amsterdam, MS Center Amsterdam, Amsterdam Neuroscience, Amsterdam, The Netherlands; Department of Neurology, Amsterdam UMC, Vrije Universiteit Amsterdam, MS Center Amsterdam, Amsterdam Neuroscience, Amsterdam, The Netherlands; Department of Clinical Neurophysiology and MEG center, Amsterdam UMC, Vrije Universiteit Amsterdam, MS Center Amsterdam, Amsterdam Neuroscience, Amsterdam, The Netherlands; University of Bordeaux, Bordeaux, France; Inserm U1215 – Neurocentre Magendie, Bordeaux, France; Department of Psychiatry and UMC Utrecht Brain Center, University Medical Center Utrecht, Utrecht University, The Netherlands; University of Bordeaux, Bordeaux, France; Inserm U1215 – Neurocentre Magendie, Bordeaux, France; CHU Pellegrin Bordeaux, Bordeaux, France

**Keywords:** Magnetoencephalography, Diffusion tensor imaging, Structural connectivity, Functional connectivity, Cognition, Multiple sclerosis

## Abstract

Multiple sclerosis (MS) features extensive connectivity changes, but how structural and functional connectivity relate, and whether this relation could be a useful biomarker for cognitive impairment in MS is unclear. This study included 79 MS patients and 40 healthy controls (HCs). Patients were classified as cognitively impaired (CI) or cognitively preserved (CP). Structural connectivity was determined using diffusion MRI and functional connectivity using resting-state magnetoencephalography (MEG) data (theta, alpha1, and alpha2 bands). Structure-function coupling was assessed by correlating modalities, and further explored in frequency bands that significantly correlated with whole-brain structural connectivity. Functional correlates of short- and long-range structural connections (based on tract length) were then specifically assessed. Receiving operating curve analyses were performed on coupling values to identify biomarker potential. Only the theta band showed significant correlations between whole-brain structural and functional connectivity (rho = −0.26, *p* = 0.023, only in MS). Long-range structure-function coupling was stronger in CI patients compared to HCs (*p* = 0.005). Short-range coupling showed no group differences. Structure-function coupling was not a significant classifier of cognitive impairment for any tract length (short-range area under the curve (AUC) = 0.498, *p* = 0.976, long-range AUC = 0.611, *p* = 0.095). Long-range structure-function coupling was stronger in CI MS compared to HCs, but more research is needed to further explore this measure as biomarkers in MS.

## Author Summary

Cognitive impairment in multiple sclerosis (MS) is common and relates to structural and functional connectivity. However, it remains unclear whether the interplay (coupling) between structural and functional connectivity could be a biomarker of MS-related cognitive impairment. This study investigated the cognitive relevance of structure-function coupling in 79 MS patients and 40 healthy controls using diffusion MRI and magnetoencephalography. Results show that coupling was stronger in cognitively impaired MS patients compared to controls, but only when considering long-distance connections. Nonetheless, classifier analyses indicated only weak biomarker potential in terms of sensitivity and specificity. Future studies should include additional operationalization of coupling as well as longitudinal and regional or network level data.

## INTRODUCTION

Patients with multiple sclerosis (MS) commonly experience deficits in cognitive performance, which profoundly affect quality of life (Chiaravalloti & DeLuca, 2008). Both structural and functional brain alterations seem to correlate with cognitive impairment in MS (Chard et al., 2021; Fleischer et al., 2019; Nauta et al., 2020). Previous studies have indicated that beside structural brain damage, such as gray matter atrophy, disruptions in both structural and functional networks are important correlates of cognitive impairment (Eijlers et al., 2018b; Faivre et al., 2016; Fleischer et al., 2019; Nauta et al., 2020). Structural and functional connectivity are commonly measured using statistical approaches and algorithms, thereby providing an estimation of true connections. Structural connectivity (SC) estimates the likelihood that white matter tracts physically interconnect brain regions based on diffusion measurements, whereas functional connectivity (FC) reflects statistical interdependencies between time series that describe activity measurements (Aertsen, Gerstein, Habib, & Palm, 1989). Interestingly, MS patients with cognitive problems may show functional network changes without severe structural damage (Eijlers et al., 2018a). How these two network modalities are related, and whether there is an important interplay between structural and functional networks that pertains to cognitive function, has rarely been studied in MS.

In healthy populations, several studies have shown a relationship between SC and FC, although varying directions of this relationship have been found: higher SC has been related to lower FC, but also vice versa (Hermundstad et al., 2013; Honey et al., 2009; Skudlarski et al., 2008). While intuitively one might expect that FC would be severely constrained by the presence of direct structural connections, previous work found strong FC without a direct structural connection (Honey et al., 2009; Robinson, 2012). Overall, in the healthy situation, the functional repertoire seems extensive despite a limited structural backbone, indicating a potentially low overlap between structure and function (van Dam, Hulst, & Schoonheim, 2021). Indeed, it has become clear that there are regional variations in SC-FC correspondence, which relate to cognition in healthy controls (HCs) (Gu, Jamison, Sabuncu, & Kuceyeski, 2021). In MS, it was found that more similarity between SC and FC (i.e., structure-function coupling) related with poorer cognitive performance (Koubiyr et al., 2020), indicating that greater correspondence between structural and functional networks of MS patients could potentially be of use as a biomarker for cognitive impairment.

Recently, it was found that particularly damage to long-range white matter tracts is important for cognitive problems in MS, which warrants a focus on the interplay between SC and FC for specifically these connections in the brain (Meijer, Steenwijk, Douw, Schoonheim, & Geurts, 2020). Our study aimed to investigate the interplay between SC and FC and its relation with cognition in MS. It was examined whether MS patients with cognitive impairment displayed a disruption of structure-function coupling and whether this effect was more apparent in specific tracts according to their length. Because structural and functional brain connectivity are inseparably connected, studying the relationship between SC and FC (i.e., coupling) could provide more sensitive and specific measures of individual response to MS-related damage compared to studying SC and FC separately, and could lead to the development of better biomarkers thereof (Honey, Thivierge, & Sporns, 2010; van den Heuvel et al., 2013).

## MATERIALS AND METHODS

### Participants

All MS patients and HCs were part of the Amsterdam MS cohort (Eijlers et al., 2018b). Subsamples of the magnetoencephalography (MEG) and diffusion magnetic resonance imaging (dMRI) data of this cohort has been published previously (Meijer et al., 2020; Nauta et al., 2020), but have never been analyzed together. In the present study participants were included who underwent cognitive assessment as well as both MEG and dMRI measurements between 2010 and 2013, resulting in the inclusion of 79 patients with a diagnosis of MS (72.2% women, age 53.77 ± 10.7 years; Table 1) and 40 HCs (62.5% women, age 50.72 ± 6.11 years; Table 1). Disability was estimated using the Expanded Disability Status Scale (EDSS) (Kurtzke, 1983). Level of education was measured on a scale of 1 (did not finish primary school) to 7 (acquired a university degree) (Verhage, 1964) and categorized as low (categories 1–4) or high (categories 5–7). Ethics approval was granted by the institutional ethics review board of the Amsterdam UMC, and written informed consent was obtained from all participants prior to participation.

**Table 1.** Demographic, clinical, cognitive, and MRI outcomes of MS patients and healthy controls

	Healthy Controls *N* = 40	MS Patients		
		Total Group *N* = 79	CP Patients *N* = 46	CI Patients *N* = 33
**Demographics**				
Age; years, mean (*SD*)	50.7 (6.11)	53.8 (10.7)	53.4 (10.8)	54.3 (10.8)
Sex; % females	63%	72%	74%	70%
Education; % low/high	40/60%	50/50%	46/54%	56/44%
**Clinical characteristics**				
Disease duration; years, mean (*SD*)	n/a	18.1 (6.93)	18.0 (6.77)	18.3 (7.3)
MS type; RR/SP/PP (%)	n/a	71/20/9%	76/17/7%	64/24/12%
EDSS; median (range)	n/a	3.50 (1–8)	3.25 (1–8)	4.00 (2.5–7.5)
**MRI characteristics**				
Cortical gray matter volume; L, mean (*SD*)	0.764 (0.033)*	0.732 (0.050)	0.740 (0.047)	0.721 (0.052)
Deep gray mater volume; mL, mean (*SD*)	61.5 (2.71)*	54.2 (7.00)	56.4 (5.58)	51.3 (7.62)
White matter lesion load; mL, median (range)	n/a	12.6 (2.47–85.5)	11.0 (3.17–61.0)	18.7 (2.47–85.5)

*Note*. Disease duration represents the disease duration since symptom onset. CI = cognitively impaired; CP = cognitively preserved; EDSS = Expanded Disability Status Scale; MS = multiple sclerosis; n/a = not applicable; PP = primary progressive; SD = standard deviation; RR = relapsing remitting; SP = secondary progressive.

* Significantly different from MS patients (*p* < 0.05).

### Magnetic Resonance Imaging

Participants were scanned on a 3T scanner (GE Signa HDxt) using an eight-channel phased-array head coil. Volumetry and registration were based on a 3D T1-weighted inversion-prepared fast spoiled gradient recall sequence (repetition time 7.8 ms, echo time 3 ms, inversion time 450 ms, flip angle 12°, sagittal 1.0-mm sections, 0.94 × 0.94 mm^2^ in-plane resolution). Lesion filling (using LEAP) was performed and deep gray matter volumes were estimated using FIRST (FSL5). SIENAX (FSL5) was used to calculate cortical gray matter volumes by masking deep gray matter areas from total gray matter segmentations. To normalize brain volumes, differences in skull size of each participant compared to the skull of the standard brain were computed by multiplying all gray matter volumes with the V-scaling factor (FSL5). SC was based on dMRI covering the entire brain using five volumes without directional weighting (i.e., b = 0 s/mm^2^) and 30 volumes with noncollinear diffusion gradients (echo planar imaging (EPI), b = 1,000 s/mm^2^, repetition time 13,000 ms, echo time 91 ms, flip angle 90°, 2.4-mm contiguous axial slices, 2 × 2 mm^2^ in-plane resolution). Automatic segmentation of hyperintense lesions was applied on FLAIR images and they were filled on the 3D T1 using LEAP (Chard, Jackson, Miller, & Wheeler-Kingshott, 2010; Steenwijk et al., 2013).

### Structural Connectivity

All dMRI preprocessing was performed as previously reported (Meijer et al., 2020), using the FMRIB Diffusion Toolbox with standard settings (FDT; part of FSL5), including brain extraction, eddy current, and motion correction. Images were then fed into MRtrix 3.0 to perform probabilistic tractography, using the fiber orientation distribution (Tournier, Calamante, & Connelly, 2012). Through this algorithm, SC in the form of number of streamlines was reconstructed by randomly putting seeds in the white matter. In order to determine possible paths (fibers) between regions, the local fiber orientation distribution was estimated using constrained spherical deconvolution (Tournier, Calamante, & Connelly, 2007). The 30 noncollinear diffusion directions in the data were adjusted by restricting the maximum spherical harmonic order (lmax) to six. Then, whole-brain probabilistic tractography was performed by randomly seeding 100 million fibers within the brain mask for each participant. Subsequently, these whole-brain maps were converted to atlas-specific maps; all connections remained unthresholded for further analyses. Cortical gray matter nodes were defined by processing the 3D T1-weighted image of each participant with the FreeSurfer 5.3 pipeline, after lesion filling (Meijer et al., 2020). The automated anatomical labeling (AAL) atlas (Tzourio-Mazoyer et al., 2002) was used to define 78 cortical nodes on the native cortical surface (Meijer et al., 2020). Subsequently, cortical regions were coregistered to dMRI space by using FLIRT (part of FSL), where MRtrix was used to visualize structural tracts between all atlas regions by using the aforementioned processed streamline data. Finally, mean fractional anisotropy (FA) was calculated and used as our measure of whole-brain SC within each tract. FA is commonly used as a measure of connectivity in the MS field (Lopez-Soley et al., 2020; Pardini et al., 2015). Importantly, the reliability of raw fiber count as a measure of SC is understudied in MS. However, concerns remain regarding the use of this approach in MS due to effects of MS pathology, which could induce false positive and/or negative connections. As such, it has been recommended that average diffusion measures (such as FA) could be a better candidate than fiber count for SC to avoid this particular issue (Lipp et al., 2020). Additionally, different types of tractography have different error types (false positive or false negatives), but tract-averaged diffusion measures were recently proposed to deal with MS-specific noise (Lipp et al., 2020). From this point, SC thus refers to the mean FA within a given tract.

### Magnetoencephalography

Eyes-closed, resting-state MEG measurements of 5 minutes were analyzed. Acquisition and preprocessing of the MEG data was performed as described previously (Derks et al., 2018). In short, measurements were performed in a magnetically shielded room (Vacuum Schmelze GmbH, Hanua, Germany) with a 306-channel MEG system (Elekta Neuromag Oy, Helsinki, Finland). Data were sampled at 1250 Hz, and a high-pass filter (0.1 Hz) and anti-aliasing filter (410 Hz) were employed online. The extended Signal Space Separation method (xSSS) (van Klink et al., 2017) was applied to facilitate visual inspection of malfunctioning channels, after which a maximum of 12 malfunctioning channels were excluded (SK, LD). Artifact removal was performed offline with the temporal extension of the SSS in MaxFilter software (Elekta Neuromag Oy, version 2.2.15) (Taulu & Simola, 2006). MEGs were subsequently coregistered with participants’ MRI using a surface-matching procedure. The outline of the scalp and four or five head localization coils were digitized and continuously monitored using a 3D digitizer (3Space Fastrak, Polhemus, Colchester, VT, USA), which was matched to the MRI scalp surface. Subsequently, the coregistered MRI was spatially normalized to a template MRI. Centroid voxels (Hillebrand et al., 2016) in the 78 cortical regions of the AAL atlas (Gong et al., 2009) were selected for further analyses after inverse transformation to the participant’s coregistered MRI. An atlas-based beamformer implementation (Elekta Neuromag Oy, version 2.1.28) was then applied to reconstruct broadband (0.5–48 Hz) time series of neural activity for these 78 centroids (Hillebrand, Barnes, Bosboom, Berendse, & Stam, 2012).

For each patient and HC, the first 13 consecutive epochs of 13.10 s (16,384 samples) were selected (Liuzzi et al., 2017). The number of included epochs was based on the participant with the lowest number of epochs available. All epochs were concatenated such that the included time series were analyzed as a whole.

### Functional Connectivity

FC was calculated for theta, alpha1, and alpha2 bands only, based on previous results showing relations with cognition in MS (Schoonheim et al., 2013; Tewarie et al., 2014a, 2014b, 2015). It should be noted that although there are papers that use the corrected amplitude envelope correlation (AECc) in HCs (Messaritaki et al., 2021), none have investigated cognition in MS, thus this choice was based on other FC metrics. Time series were therefore filtered in the theta (4–8 Hz), alpha1 (8–10 Hz), and alpha2 (10–13 Hz) bands by digital band-pass filtering using a fast Fourier transform, after which all bins outside the pass bands were set to zero, and an inverse Fourier transform was performed.

To estimate FC between time series of each pair of AAL regions, the AEC (Brookes et al., 2011; Hipp, Hawellek, Corbetta, Siegel, & Engel, 2012) was calculated. The AEC measures amplitude-based connectivity between each pair of brain regions, based on correlations between their amplitude envelopes. To calculate the AEC, the Hilbert transform was performed on the band-pass filtered time series. Subsequently, since source-reconstructed MEG data is contaminated by signal leakage (Stam, Nolte, & Daffertshofer, 2007), the AEC was computed after pairwise orthogonalization of time series in the time domain, resulting in the corrected AEC (AECc). To avoid negative values in the FC matrices, values were rescaled according to $\frac{\text{AECc}+1}{2}$. FC was calculated in Matlab (version 2018.b, Mathworks, Natick, MA, USA) using in-house scripts. Whole-brain FC was calculated by averaging the FC matrices over all regions.

### Short- and Long-Range Connections

**Figure 1.**
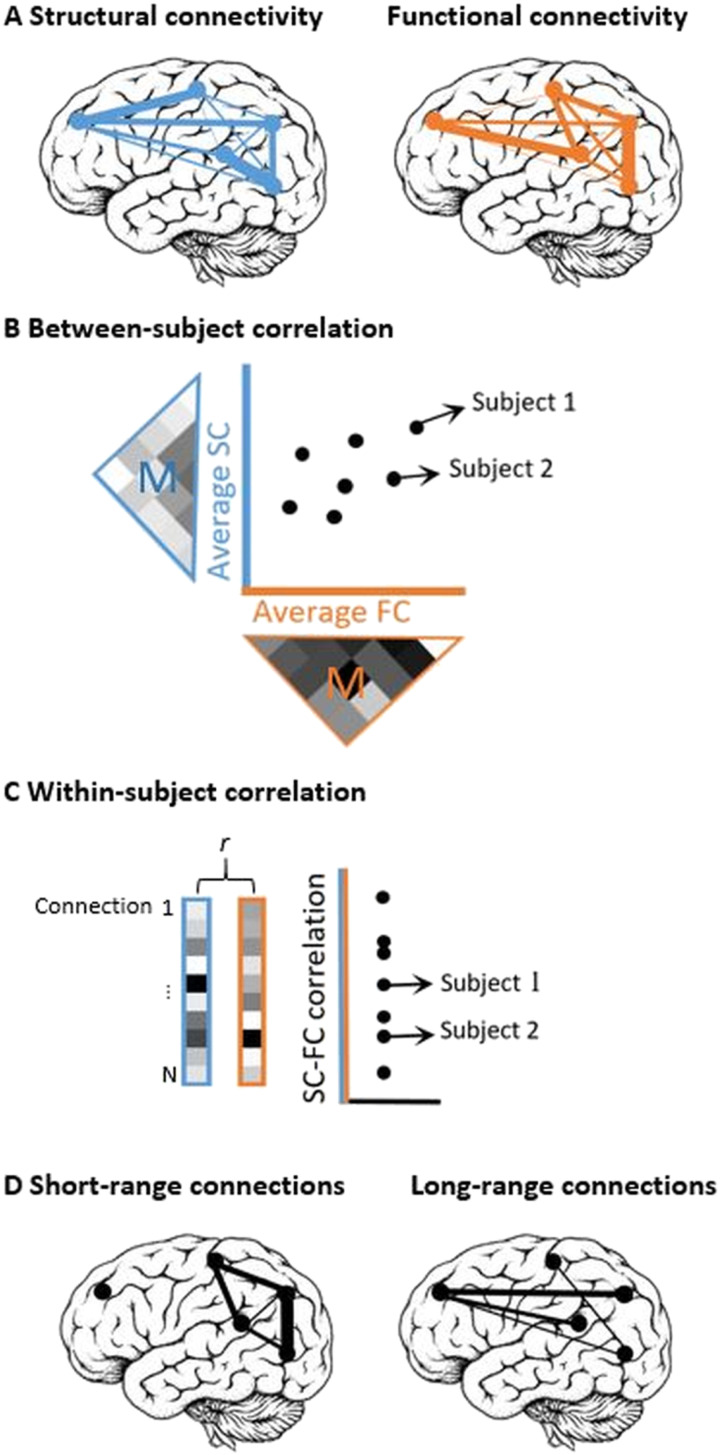
Overview of the applied methods to calculate structure-function relationships. (A) Schematic representation of SC and FC. (B) Between-subject correlations were calculated by first averaging all connections in the upper triangle of each individual subject’s matrix for both SC (blue M) and FC (orange M). Subsequently, these averaged values for SC and FC were correlated across subjects within the HC and MS groups separately. (C) Within-subject correlations were calculated by first vectorizing all short- and long-range connections (see panel D) in the upper triangle of each subject’s matrix for both SC and FC. Second, these SC and FC vectors were correlated within each subject to determine structure-function coupling. (D) Schematic representation of short- (Q1) and long-range (Q4) connections, based on the first and fourth quartiles of the histogram of tract lengths in HCs. SC = structural connectivity; FC = functional connectivity; M = mean, Q1 = first quartile; Q4 = fourth quartile.

**Figure 2.**
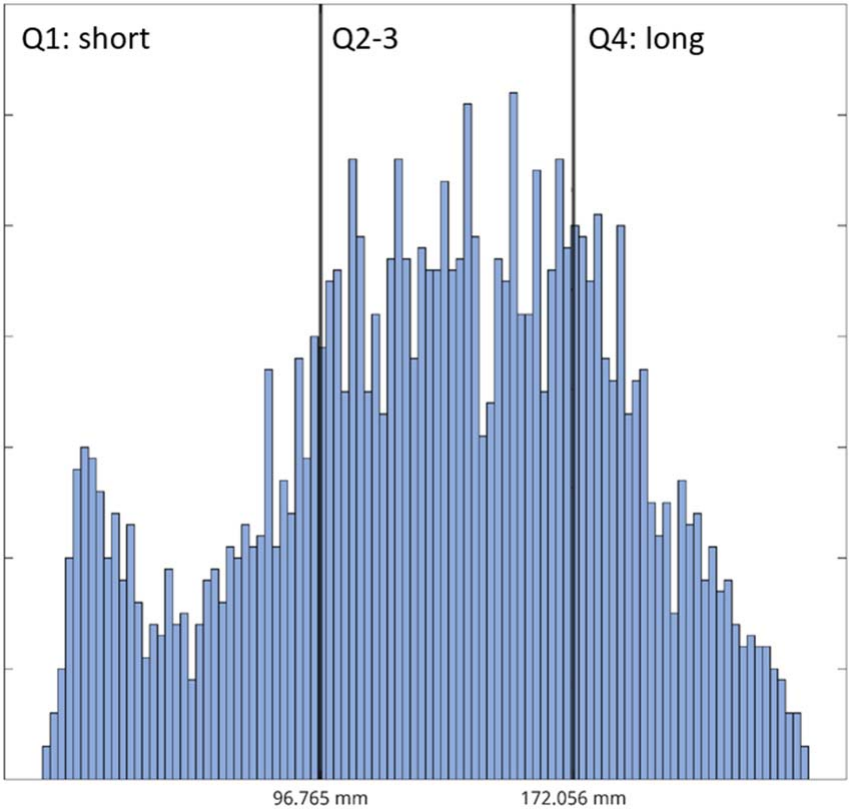
Distribution of tract lengths in healthy controls. Structural connections of both HCs and MS patients were categorized into short-range (<96.765 mm) and long-range connections (>172.056 mm) based on first and fourth quartile thresholds. Q1 = first quartile; Q2 = second quartile; Q3 = third quartile; Q4 = fourth quartile.

Short- and long-range connections of both SC and FC matrices were determined as previously described (Meijer et al., 2020). Structural connections were divided into short- (first quartile (Q1), <96.765 mm) and long- (fourth quartile (Q4), >172.056 mm) connections (see Figure 1D), based on the histogram of tract lengths of HCs (see Figure 2), as calculated on dMRI by MRtrix. Subsequently, to determine short- and long-range FC, only functional connections with a direct underlying structural connection (i.e., short- or long-range connection) were taken into account.

### Structure-Function Relationships

Figure 1 represents an overview of how the structure-function relationships were constructed. Between-subject correlations (Figure 1B): To determine the group-level correlation between mean SC and mean FC within HCs and MS, SC and FC were first averaged for each subject across all connections in the upper triangles of the respective matrices. Subsequently, this averaged SC value and averaged FC value per subject were correlated across all subjects in the MS and HC groups separately, using Pearson’s correlation coefficients. This approach therefore provided one correlation coefficient per group indicating how individual differences in global functional and SC are related. Between-subject correlations were calculated for whole-brain, short-, and long-range connections.

The within-subject correlations (Figure 1C) measure of structure-function coupling was calculated for each individual subject by first vectorizing the short- and long-range connection weights of both SC and FC matrices within each participant (i.e., the short- and long-range connections weights in the upper triangle of the matrix were transformed into one column). Second, these SC and FC vectors were correlated within each participant using Pearson’s correlations, resulting in one structure-function coupling value per participant for both short- and long-range connections. This measure therefore indicates whether connectivity weights correlate across the structural and functional network of an individual.

### Neuropsychological Evaluation

Neuropsychological assessment was based on an expanded Brief Repeatable Battery of Neuropsychological tests (BRB-N), as described previously (Eijlers et al., 2018b). The assessment consisted of seven neuropsychological tests: (1) the Selective Reminding Test (verbal memory); (2) the 10/36 Spatial Recall Test (visuospatial memory); (3) the Symbol Digit Modalities Test (information processing speed); (4) the paper and pencil Memory Comparison Test (working memory); (5) the Word List Generation Test (semantic verbal fluency); (6) the Concept Shifting Test (executive function); and (7) the Stroop Color-Word Test (attention and executive function). Details on the raw test scores that have been used were described previously (Eijlers et al., 2018b). Based on a normative sample of HCs, the raw test scores were adjusted for age, sex, and education, as described previously (Amato et al., 2006; Eijlers et al., 2018b). These adjusted scores were converted into *z*-scores based on the means and standard deviations of the HCs and subsequently averaged into test-specific *z*-scores.

The MS patients were categorized as cognitively impaired (CI; 2 *SD*s (i.e., *z* ≤ −2) below the average of the HCs on at least two cognitive domains) or cognitively preserved (CP; remainder).

### Classification Analyses

Receiving operating curve (ROC) analyses were performed to determine whether structure-function coupling in whole-brain, short-, and long-range connections could classify CI patients among MS patients. The areas under the curve (AUCs) were reported and optimal cutoff scores (i.e., the highest value for sensitivity and specificity combined) were defined.

### Statistical Analyses

Statistical analyses were performed in SPSS 26.0 (Chicago, IL, USA) and in Matlab. All outcome measures were checked for normal distributions using histogram inspection.

To test whether the sparsity of the SC matrices differed between groups, which could potentially have affected subsequent analyses, the sparsity of the SC matrices was calculated per subject. Subsequently, these sparsity values were compared between HCs, CP, and CI MS patients with a general linear model. Additionally, the number of short- and long-range connections were compared between MS patients and HCs, also with a general linear model.

Subsequently, to calculate between-subject correlations, relationships between average whole-brain SC and FC were quantified in MS and HCs separately using Pearson’s correlations. Only those frequency bands in which a significant relation between SC and FC was found in either MS or HCs (or both) were explored further to limit the number of statistical comparisons.

Then, to calculate between-subject correlations for short- and long-range connections, Pearson’s correlations between average short-range SC and FC and long-range SC and FC were performed within identified bands, using the same approach within MS patients and HCs.

Next, the clinical relevance of within-subject coupling, that is, short- and long-range coupling, was explored by comparing these between HCs, CP, and CI with general linear models, correcting for age and sex. When significant group effects were found, it was investigated which groups differed significantly. Coupling measures that significantly differed between groups were further explored by correlating them with cognitive subdomains and disability, as well as volumes of lesions, deep and cortical gray matter, using Pearson’s or Spearman’s (if not normally distributed) correlations.

Finally, between-group differences of SC and FC separately in the previously determined frequency bands were assessed with general linear models, correcting for age, sex, and education.

Significance level was set at *p* < 0.05. Analyses including short- and long-range connections were Bonferroni corrected for multiple comparisons by dividing the *p* value by two (*p* < 0.025), and group comparisons were Bonferroni corrected by dividing the *p* value by three (three group comparisons; *p* < 0.017).

### Post Hoc Analyses

To investigate the specificity of our results, a post hoc analysis was performed calculating between-subject correlations for both short- and long-range connections in the other frequency bands. Additionally, it was assessed whether relative power of the previously selected frequency bands was correlated to SC, as the more basic measure of power could confound the relationship between SC and FC. Furthermore, because the division of tracts into short- and long-range was previously only performed for structural connections (Meijer et al., 2020), it was further investigated, in post hoc analyses, whether this division was also applicable to functional connections. To test the distinctiveness of short- and long-range FC, short-range FC was correlated with long-range SC, and vice versa. Additionally, whole-brain FC theta was correlated to both FC theta of short- and long-range connections within the MS patients.

## RESULTS

### Characteristics of Included Participants

Patients did not differ from HCs with regard to age, sex, and level of education (*p* > 0.05). Table 1 presents an overview of all demographic and clinical variables. The patient cohort was moderately affected based on disability (median EDSS 3.5), with an average disease duration of 18 years (range 8.83–37.7). Average cognitive performance was significantly lower in MS patients compared to HCs (*p* < 0.001), with 33 (42%) patients displaying cognitive impairment. No significant difference was found in the sparsity of the SC matrices between HCs, CI, and CP MS patients (*F* = 0.773, *p* = 0.464). Also, there was no difference between the number of short-range (mean: 1,628.9 for HCs, 1,707.2 for MS, *F* = 3.023, *p* = 0.085) and long-range (mean: 1,591.8 for HCs, 1,534.3 for MS, *F* = 0.193, *p* = 0.662) connections when comparing MS patients with HCs.

### Between-Subject Correlations: Relationships Between SC and FC

**Figure 3.**
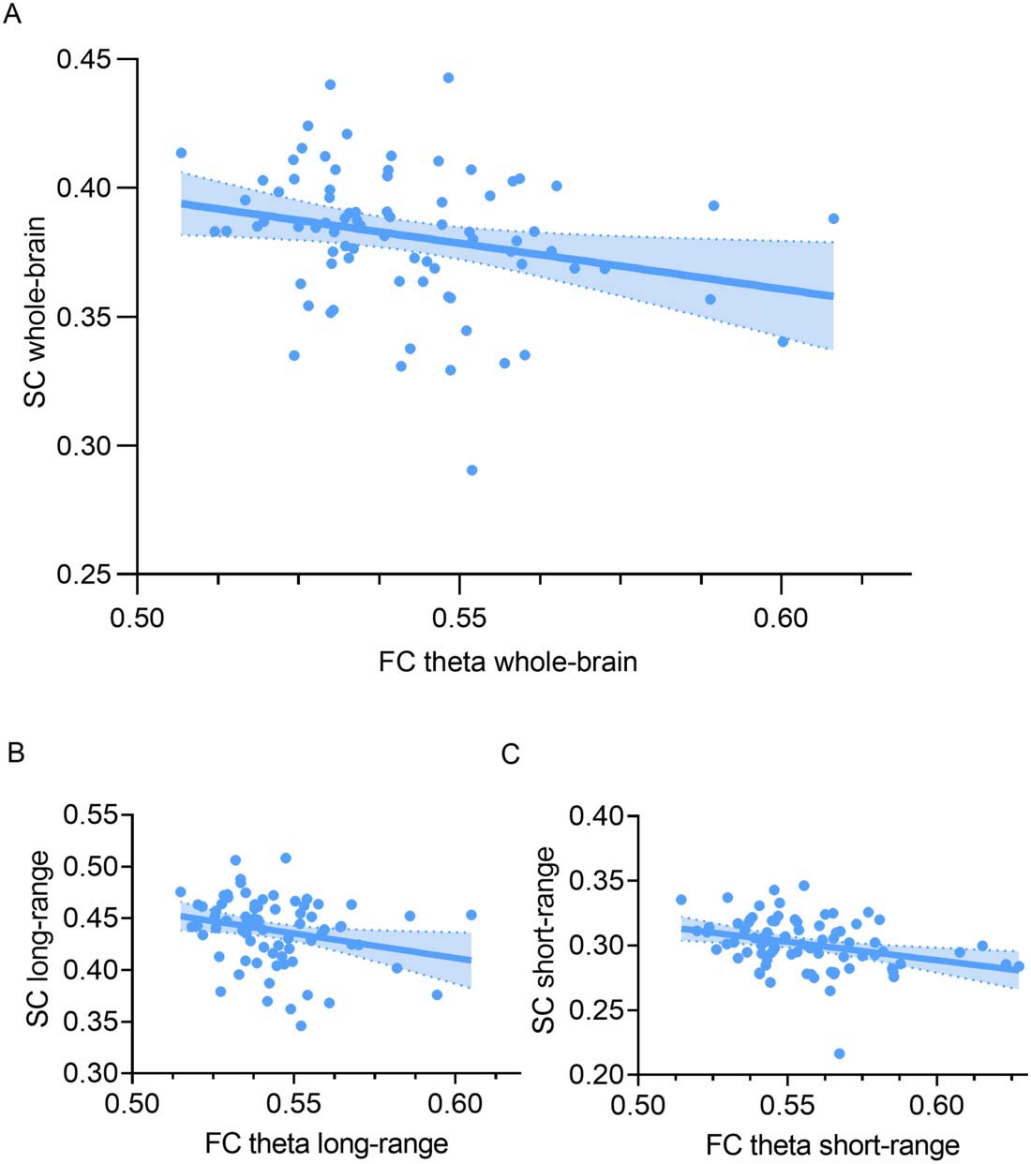
Between-subject correlations: the relationship between FC theta and SC in MS. (A) Relation between whole-brain SC and FC (*r* = −0.256, *p* = 0.023). (B) Relation between average long-range SC and FC (*r* = −0.248, *p* = 0.028). (C) Relation between average short-range SC and FC (*r* = −0.313, *p* = 0.005). SC = structural connectivity; FC = functional connectivity.

Within MS, whole-brain SC was significantly related to whole-brain FC in the theta band only (*r* = −0.256, *p* = 0.023; Figure 3), which was not significant in HCs (*r* = −0.061, *p* = 0.711). Whole-brain FC in the alpha bands did not show significant correlations with SC in either group (MS patients alpha1: *r* = −0.090, *p* = 0.429, alpha2: *r* = −0.097, *p* = 0.394, HCs alpha1: *r* = −0.118, *p* = 0.467, alpha2: *r* = −0.012, *p* = 0.940), thus only the theta band was further explored.

Correlations between average short-range SC and average short-range FC theta were significant in MS (*r* = −0.313, *p* = 0.005; Figure 3), but not in HCs (*r* = −0.172, *p* = 0.290). For long-range connections there was also a significant relation between average SC and average FC theta in MS (*r* = −0.248, *p* = 0.028, not significant after correcting for two tests performed; Figure 3), but not in HCs (*r* = −0.068, *p* = 0.675). As such, both short- and long-range coupling in the theta band were evaluated further.

### Within-Subject Correlations: Structure-Function Coupling

**Figure 4.**
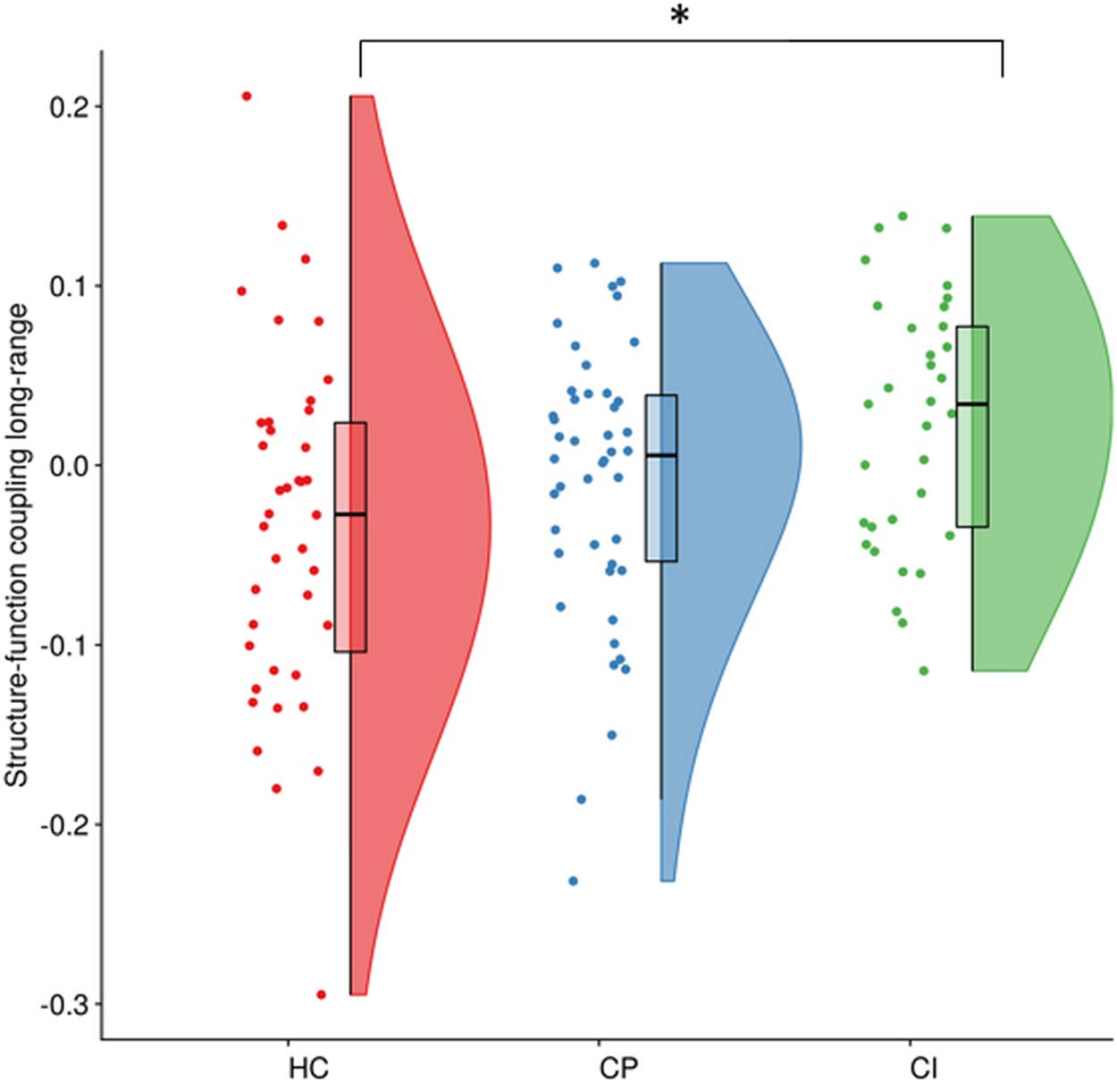
The structure-function coupling for long-range connections within the different groups. Each dot denotes a participant. Boxplots show the median value per group; *p < 0.05. HC = healthy controls; CP = cognitively preserved MS patients; CI = cognitively impaired MS patients.

A significant effect of group for long-range structure-function coupling (*F* = 4.04, *p* = 0.020; significant after correcting for two tests performed) was found, which was driven by an increase in CI (*M* = 0.022, *SD* = 0.014) compared to HCs (*M* = −0.033, *SD* = 0.013) (*p* = 0.005; significant after correcting for three group comparisons; Figure 4), but not between HCs and CP (*p* = 0.163), or CP and CI (*p* = 0.109). No significant group effects were seen for short-range coupling (*F* = 0.025, *p* = 0.975), which was not explored further.

### Structure-Function Coupling and Clinical Scores

Within MS, correlations with cognitive subdomains, clinical disability, and MR measures were only performed for long-range coupling values, as this coupling value significantly differed between HCs and CI MS patients. All performed correlations were not significant (executive functioning: *r* = 0.002, *p* = 0.884; verbal memory: *r* = 0.018, *p* = 0.891; information processing speed: *r* = −0.129, *p* = 0.328; verbal fluency: *r* = 0.074, *p* = 0.571; visuospatial memory: *r* = −0.012, *p* = 0.928; disability: *r* = 0.051, *p* = 0.660; lesion volume: *Rho* = −0.007, *p* = 0.960; and atrophy: normalized deep gray matter volume: *r* = −0.009, *p* = 0.947; normalized cortical gray matter volume: *r* = −0.049, *p* = 0.708).

### Comparisons Within Long-Range Connections

**Figure 5.**
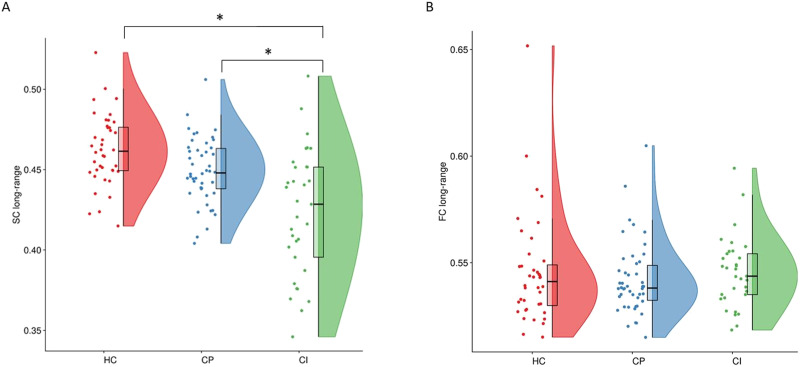
SC and FC of long-range connections across groups. Each dot denotes a participant. Boxplots show the median value per group. (A) SC of long-range connections per group. (B) FC of long-range connections per group. *p < 0.05. SC = structural connectivity; FC = functional connectivity; HC = healthy controls; CP = cognitively preserved MS patients; CI = cognitively impaired MS patients.

Finally, differences in SC and FC theta of long-range connections were separately assessed between groups. Long-range SC showed a significant effect of group (*F* = 15.6, *p* < 0.001), with CI (*M* = 0.423, *SD* = 0.005) showing lower values compared to both CP (*M* = 0.449, *SD* = 0.004, *p* < 0.001) and HCs (*M* = 0.461, *SD* = 0.005, *p* < 0.001; Figure 5). Conversely, FC theta of long-range connections showed no effect of group (*F* = 0.130, *p* = 0.878; Figure 5).

### Classification Analyses

Receiving operating curve (ROC) analyses showed that structure-function coupling was not a significant classifier of cognitive impairment among MS patients, neither for whole-brain (AUC = 0.493, *p* = 0.913), short-range (AUC = 0.498, *p* = 0.976), or long-range connections (AUC = 0.611, *p* = 0.095). Optimal cutoff scores for structure-function coupling was −0.03 for whole-brain connections (sensitivity = 15%, specificity = 93%), −0.03 for short-range connections (sensitivity = 49%, specificity = 65%), and 0.04 for long-range connections (sensitivity = 46%, specificity = 80%).

### Post Hoc Analyses

To assess the specificity of our results, SC and FC were also correlated in the alpha1 and alpha2 bands, showing a significant correlation between SC and FC of short-range connections in the alpha1 band for the MS patients (rho = −0.271, *p* = 0.016, significant after correcting for two tests performed), but not for HCs (rho = −0.106, *p* = 0.514). Long-range connections showed no correlations (*p* < 0.05; see Table 2 for all results). Next, the relative power in the theta band was correlated to whole-brain SC in MS, yielding nonsignificant results (*r* = −0.176, *p* = 0.121), which indicates that the relationship between SC and FC is not likely to be driven by power.

**Table 2.** Between-subject correlations for short- and long-range connections

	MS	HC
Short-range alpha1	*r* = −0.271, *p* = 0.016	*r* = −0.106, *p* = 0.514
Short-range alpha2	*r* = −0.193, *p* = 0.089	*r* = 0.041, *p* = 0.803
Long-range alpha1	*r* = −0.017, *p* = 0.880	*r* = −0.160, *p* = 0.323
Long-rang alpha2	*r* = −0.096, *p* = 0.400	*r* = −0.096, *p* = 0.554

Furthermore, significant correlations were found between FC theta of short-range connections and SC of long-range connections: *r* = −0.280, *p* = 0.005 (significant after correcting for two tests performed) and FC theta of long-range connections and SC of short-range connections: *r* = −0.330, *p* = 0.003 (significant after correcting for two tests performed). When further zooming in on FC, whole-brain FC theta showed a strong correlation to FC theta of both short- (*r* = 0.990, *p* < 0.001, significant after correcting for two tests performed) and long-range (*r* = 0.986, *p* < 0.001, significant after correcting for two tests performed) connections within the MS patients, indicating that short- and long-range connections within the functional network may not be as distinctive as they are in the structural network.

## DISCUSSION

This study aimed to investigate the cognitive relevance of altered coupling between SC and FC in MS. Significant correlations between SC and FC were only seen in MS but not in HCs, and only in the theta band. Coupling of FC theta and SC was stronger in CI MS patients compared to HCs, which was specific for long-range connections.

Between-subject correlations showed that SC was (negatively) related to FC in the theta band in MS, indicating that patients with more structural damage have higher FC. Previously, it has been shown that such ‘hyperconnectivity’ is common in neurological diseases as a reaction to structural damage (Hillary et al., 2015; Schoonheim, Meijer, & Geurts, 2015). The theta band is typically related to relaxed wakefulness (Mari-Acevedo, Yelvington, & Tatum, 2019). In MS, the theta band has been described before, showing increased power and FC in relation to cognitive impairment (Schoonheim et al., 2013; Schoonhoven et al., 2019; Tewarie et al., 2015; Van der Meer et al., 2013). Why specifically this band would show a relationship between SC and FC in MS remains unclear. Possibly, structural damage in the form of lesions, which reduces SC, could result in the previously observed increased FC in the theta band. Such increased FC in the theta band was also seen in other neurological disorders such as Alzheimer’s disease, albeit using a different connectivity measure (Briels et al., 2020). Also, this relationship was found for whole-brain and short-range connections regarding SC and FC theta, and for short-range connections regarding SC and FC alpha1, whereas in the long-range connections this relationship did not survive corrections for multiple comparisons. Additionally, the between-subject correlation between SC and FC was only found in MS patients and not in HCs. Correlations between SC and FC have previously also been found in HCs using both MEG and fMRI (Hermundstad et al., 2013; Honey et al., 2009; Meier et al., 2016; Skudlarski et al., 2008; Tewarie et al., 2019). This specificity to the MS group could be due to MS pathology itself, either because MS changes the relationship between SC and FC, or because MS has an effect on SC and FC separately, or both. At the same time, methodological issues may have obscured correlations between SC and FC in our HCs. As it has been suggested that different methods to quantify FC lead to different relationships between SC and FC it seems plausible that using a different imaging modality could lead to different findings in this relationship (Liegeois, Santos, Matta, Van De Ville, & Sayed, 2020). Also, perhaps the small control sample that was included in our study might have influenced the statistical power to determine a significant correlation coefficient within these HCs. As such, future work remains needed to confirm these specific results.

When investigating CP and CI MS patients separately in comparison to HCs, long-range coupling (i.e., within-subject coupling) was stronger in CI, indicating a stronger overlap in structural and functional networks in CI patients compared to HCs. This finding is in line with previous work where it was shown that a lower overlap between SC and FC is related to better cognitive performance (Wang et al., 2018), which is further supported by a study in dementia patients in which also a stronger relationship between SC and FC was found (Cao et al., 2020). This specific effect in CI could be explained by the higher density of short-range compared to long-range structural connections in the brain, leading to an increased vulnerability of long-range connections (Park & Friston, 2013). Thus, alterations to long-range connections may have larger consequences on the functional network, limiting the repertoire of functional possibilities when these connections are damaged (van Dam et al., 2021). This limited repertoire would then result in stronger coupling, which has longitudinally been observed in a previous MS study (Koubiyr et al., 2020). On the other hand, long-range coupling showing higher values in CI patients compared to HCs could also be explained by the relationship between long-range structural connections and cognitive performance only. Moreover, a recent study found specifically that damage of long-range structural connections was related to cognitive impairment in MS patients (Meijer et al., 2020). As our analyses indicated that short- and long-range connections in the functional network may not be similarly distinctive as they are in the structural network, and because this division is based on structural tracts, it might not be applicable to FC. In fact, the present study did not identify group differences in theta band long-range FC, while previous research has indicated that theta band whole-brain network topology is altered in MS using MEG, albeit using a different FC measure (Nauta et al., 2020). In addition, there was no relation between long-range coupling and individual cognitive domains and disability. Previous work did find a relationship between whole-brain structure-function coupling and clinical disability (EDSS score) (Koubiyr et al., 2020). Of note, the aforementioned study used fMRI instead of MEG to calculate FC and only included MS patients in the early stages of the disease, whereas patients with a wide range of disease durations were included in our study. Also, relatively low correlational values (see Figure 3) were obtained between whole-brain and short- and long-range SC and FC. Previously mentioned technical points could be the reason why our results indicate that structure-function coupling as operationalized here is not a relevant biomarker for cognitive impairment in MS. Although we have observed interesting between-group differences, our AUC analyses indicate that the biomarker potential of this quantification of “coupling” remains low, at least when assessing cross-sectional measures. Future work is needed to investigate whether this measure could be used to predict subsequent cognitive decline in MS patients. This is supported by recent MEG work from our group on cognitive functioning in MS, indicating that cross-sectional correlates of cognition can differ from longitudinal predictors (Nauta et al., 2020). Additionally, it was recently shown that regional SC-FC coupling might be a more specific and sensitive measure with regard to its relation with cognitive performance (Gu et al., 2021). Therefore, including regional information using additional functional modalities might yield more useful biomarkers.

This study does have some limitations. First, more research into the comparison between FA and the number of streamlines is lacking and newer diffusion sequences and pipelines could result in improvement in streamline quantifications. Second, the high correlation between short-range and long-range FC might be related to how FC was quantified. The AECc was applied to estimate FC and is a measure that has not been applied to MS data before. This measure was chosen because of its consistency in replicating group differences in other patient populations (Colclough et al., 2016), and has been utilized in many previous studies (Brookes et al., 2011; Tewarie et al., 2016). However, it may be insensitive to the specific relevance of short- and long-range structural connections. Our FC measure may also have been insensitive due to its pairwise nature. It is now also possible to determine FC by incorporating more than two brain regions, that is, higher order interactions (Suarez, Markello, Betzel, & Misic, 2020). Because it is known that SC and FC are not perfectly aligned, models of higher order interactions might contribute to a better understanding of FC (Suarez et al., 2020), and subsequently of the structure-function relationship. An additional important methodological issue in this study is that only functional connections with an underlying structural tract were taken into account. Importantly, the functional connections that were therefore not included in our analyses could have been involved in cognitive impairment.

To conclude, our results indicate that SC and FC are more strongly related in MS patients than in HCs, perhaps indicating a loss of the functional repertoire due to structural damage. Additionally, structure-function coupling of only long-range connections was stronger in CI MS patients, although the functional relevance of anatomical distance remains unclear. Future longitudinal work is required to further investigate regional disease stage-specific changes in structure-function coupling in MS.

## ACKNOWLEDGMENTS

We would like to thank all patients and healthy controls for their participation.

## AUTHOR CONTRIBUTIONS

Shanna Kulik: Conceptualization; Data curation; Formal analysis; Investigation; Methodology; Software; Visualization; Writing – original draft; Writing – review & editing. Ilse Nauta: Conceptualization; Data curation; Formal analysis; Investigation; Methodology; Visualization; Writing – original draft; Writing – review & editing. Prejaas Tewarie: Data curation; Project administration; Writing – review & editing. Ismail Koubiyr: Conceptualization; Writing – review & editing. Edwin van Dellen: Writing – review & editing. Aurelie Ruet: Conceptualization; Writing – review & editing. Kim Meijer: Conceptualization; Writing – review & editing. Brigit de Jong: Funding acquisition; Writing – review & editing. Cornelis Stam: Data curation; Writing – review & editing. Arjan Hillebrand: Data curation; Software; Writing – review & editing. Jeroen Geurts: Conceptualization; Funding acquisition; Supervision; Writing – review & editing. Linda Douw: Conceptualization; Data curation; Investigation; Methodology; Supervision; Writing – original draft; Writing – review & editing. Menno Schoonheim: Conceptualization; Data curation; Funding acquisition; Investigation; Methodology; Supervision; Writing – original draft; Writing – review & editing.

## FUNDING INFORMATION

Brigit de Jong, Stichting MS Research (https://dx.doi.org/10.13039/501100003000), Award ID: 15-911. Jeroen Geurts and Menno Schoonheim, Stichting MS Research (https://dx.doi.org/10.13039/501100003000), Award ID: 14-358e.

## TECHNICAL TERMS

Structural connectivity: Estimation of white matter tracts that physically interconnect brain regions based on diffusion measurements.
Functional connectivity: Statistical interdependencies between time series that describe activity measurements.
Biomarker: An indicator of a biological state (e.g., disease) or process that can be measured via various means.
MEG: Magnetoencephalography; recording the magnetic fields produced by electrical currents generated by neural populations.
dMRI: Specific MRI sequence that quantifies the diffusion of water molecules in different directions, which can be used for probabilistic tractography.
Tractography: Method for tracking the trajectory of the axonal pathways that exploits the anisotropy of the diffusion MRI signal.
Fractional anisotropy: Scalar value between zero and one that describes the degree of anisotropy of a diffusion process.
